# Impact of GLP-1 Receptor Agonists on Cognitive Performance in Adults at Risk of Alzheimer’s Disease: A Systematic Review and Meta-Analysis of Randomized Controlled Trials

**DOI:** 10.3390/nu18203312

**Published:** 2026-10-09

**Authors:** Angela M. Abbatecola, Giuseppe Migliara, Fausto D’Agostino, Nicola Veronese, Angelica Giuliani, Salvatore Caponnetto, Graziamaria Corbi, Elisabetta Signoriello, Raffaele Antonelli Incalzi, Giuseppe Paolisso

**Affiliations:** 1 Department of Life Sciences, Health, and Health Professions, Link Campus University, Via del Casale Pio V, 00165 Rome, Italy; 2 Department of Anaesthesia, Intensive Care and Pain Management, Campus Bio Medico University and Teaching Hospital, 00128 Rome, Italy; 3 Faculty of Medicine, Saint Camillus International University of Health Sciences, 00131 Rome, Italy; 4 Clinic of Laboratory and Precision Medicine, IRCCS INRCA, 60121 Ancona, Italy; 5 Department of Clinical and Molecular Sciences, Università Politecnica delle Marche, 60100 Ancona, Italy; 6 Department of Medical and Translational Medicine, University of Naples “Federico II”, 80131 Naples, Italy; 7 Department of Advanced Medical and Surgical Sciences, University of Campania Luigi Vanvitelli, 80138 Naples, Italy; 8 Research Unit of Geriatrics, Università Campus Bio-Medico di Rome, 00128 Rome, Italy; 9 Operative Research Unit of Internal Medicine, Fondazione Policlinico Universitario Campus Bio-Medico, 00128 Rome, Italy

**Keywords:** nutrition, glucagon-like peptide-1 agonists, memory, T2DM, mild cognitive impairment, RCTs

## Abstract

**Background/Objectives:** Type 2 diabetes, a widely recognized risk factor of Alzheimer’s disease (AD), is mainly due to the role played by insulin resistance on neuronal degeneration. Mild Cognitive Impairment (MCI) also significantly increases the risk of developing AD by approximately 40%. Epidemiological studies have demonstrated correlations between glucagon-like peptide-1 agonists (GLP-1RAs) on reducing the risk of AD. We synthesize current data from randomized controlled trials (RCTs) to summarize the effects of GLP-1RA treatment in adults with Type 2 diabetes or with MCI on cognitive performance. **Methods:** We searched databases from inception to 1 January 2026 investigating effects of GLP-1RA(s) on cognition. PubMed, Web of Science and Scopus databases were explored following Preferred Reporting Items for Systematic Reviews and Meta-Analyses (PRISMA). We selected only RCTs in adults investigating GLP-1RA(s) on cognitive performance. **Results:** Overall mean age was 64.5 yrs with an average follow-up period of 28.4 months. A total of 13,711 participants from five RCTs were included. Following a source-data audit, four randomized controlled trials contributed analytically compatible data to at least one cognitive domain. Cognitive domains were analyzed separately using Hedges’ g. Random-effects models used restricted maximum likelihood (REML) estimation with Knapp–Hartung inference when at least two compatible studies contributed. For global cognition, three studies yielded g = 0.014 (95% CI: −0.061 to 0.088; *p* = 0.515; *I*^2^ = 0%). For attention, two studies yielded g = 0.072 (95% CI: −2.164 to 2.308; *p* = 0.753; *I*^2^ = 31.1%). For delayed memory, executive function, and verbal function, only one compatible study was available per domain; these were therefore reported as single-study estimates rather than pooled effects. A sensitivity analysis excluding the dominant Cukierman trial yielded an imprecise and non-significant effect estimate for global cognition (g = −0.205, 95% CI: −2.678 to 2.269) indicating that the robustness of the pooled result could not be established. **Conclusions:** Current randomized evidence is insufficient to establish a consistent beneficial effect of GLP-1RA(s) on cognitive function in adults at risk of AD. The available findings should be considered exploratory and hypothesis-generating because few analytically compatible trials were available within each cognitive domain.

## 1. Introduction

Alzheimer’s disease (AD) currently burdens over 55 million people on a worldwide scale and is predicted to triple by 2050 [1]. Type 2 diabetes mellitus has been shown to negatively affect cognitive performance and is associated with an approximately twofold increased risk of AD [2], with chronic hyperglycemia and insulin resistance potentially contributing to neuronal dysfunction and accelerated cognitive decline [3,4]. Insulin plays a pivotal role in normal cognitive functioning and directly regulates brain glucose metabolism. Insulin resistance and altered insulin/insulin growth factor (IGF) signaling have been implicated in reduced cognitive performance [5,6], as well as in AD’s main etiopathogenic cascades, namely beta-amyloid (AB) deposition and tau protein hyperphosphorylation [7]. In addition to insulin resistance, other mechanisms implicated in the development of AD include neuro-inflammation, oxidative stress, mitochondrial dysfunction, and vascular dysfunction.

Given these shared metabolic and molecular pathways, the potential of glucose-lowering therapies that specifically improve insulin sensitivity as disease-modifying strategies for cognitive decline has emerged as a growing area of research [8]. Preclinical experimental models and AD clinical trials suggest that selected antidiabetic agents may exert neuroprotective effects [9]. Among these, glucagon-like peptide-1 agonists (GLP-1RA(s)) have gained increasing attention due to numerous epidemiological studies reporting associations between GLP-1RA and a reduced risk of dementia, thus raising interest in potential cognitive benefits beyond glycemic control [10]. Considering their neurotrophic, neuroprotective actions, GLP-1RA(s) are evolving as potential disease-modifying treatments even in those without Type 2 diabetes, who are afflicted with early AD and Mild Cognitive Impairment (MCI) [11,12,13]. GLP-1RA(s) not only improve insulin sensitivity but also exhibit anti-inflammatory and antioxidant properties that may attenuate neuro-inflammation [14,15]. In addition, GLP-1RA(s) can cross the blood–brain barrier and thus, may directly improve central insulin signaling [16].

MCI also significantly increases the risk of developing AD by approximately 40% [17]. Cognitive impairment in individuals at risk of AD commonly involves specific areas related to deficits in memory, attention, verbal abilities, and executive functioning. Neuropsychological testing of these domains may reflect metabolic and neurofunctional status of specific brain areas and may be sensitive to therapeutic interventions targeting insulin resistance. At the moment, there is a need to recognize adults at a high risk of developing AD and test the potential impact of GLP-1RA(s) on cognitive performance.

Given this background, the present systematic review and meta-analysis aimed at pooling evidence from available randomized controlled trials (RCTs) to evaluate the effects of GLP-1RA(s) on specific cognitive outcomes in adults at high risk of developing AD (Type 2 diabetes or MCI).

## 2. Materials and Methods

### 2.1. Search Strategy

We performed a systematic review with meta-analysis, in accordance with the Cochrane Collaboration guidelines, and reported the findings following the Preferred Reporting Items for Systematic Reviews and Meta-Analyses (PRISMA) guidelines [18]. A protocol was preregistered on PROSPERO (number: CRD420261301187) and accessed at https://www.crd.york.ac.uk/PROSPERO/view/CRD420261301187 (accessed 5 March 2026) [19].

Three electronic bibliographic databases (PubMed, Scopus and Web of Science) were searched from inception to 1 January 2026 with the search strings adapted to each database’s syntax.

Key terms included combinations of diverse condition specific terms (e.g., *“glucagon-like peptide-1 receptor agonists”, “glp-1 agonist”, “glp-1ra”, “glp-1 analog”, “incretin mimetic” “lixisenatide”, “liraglutide”, “semaglutide”, “albiglutide”, “dulaglutide”, “exenatide” and “Dementia”, “Cognitive impairment”, “MCI”, “Neurocognitive disorder”, “Cognitive decline”, “Memory disorder”, “Memory impairment”, “Cognitive dysfunction”, “Brain degeneration”, “Mental deterioration”, “Alzheimer”, “MMSE”, “Mini Mental State Examination”, “neuropsychological test battery”, “NTB”, “neurobehavioral test battery”, “neuropsychological evaluation”, “neuropsychological assessment”, “GP-COG”, “assessment of cognition”, “evaluation of cognition”, “cognitive assessment”, “cognitive evaluation”, “cognitive test”, “short test of mental status”, “STMS”, “short portable mental status questionnaire”, SPMSQ”, “Pfeiffer test”, “clock drawing test”, “CDT”, “MDB”, “mental deterioration battery”, “FAB”, “frontal assessment battery”, “ADAS-Cog”, “AD assessment scale-cognition”, “Digit span”, “NCSD”, “BPSD”, “global deterioration scale”, “neuropsychological tests”).* The search strategies detailed by database are reported in Supplementary File S1.

### 2.2. Hand Search Was Conducted by Reviewing the Bibliographies of Included Studies

We built the search string following this PICO question: P—adults over the age of 18 years with Type 2 diabetes or with MCI (no Type 2 diabetes); I—RCT testing treatment with GLP-1RA; C—compare treatment with GLP-1RA to placebo or other anti-diabetic agents; O—what is the effect of GLP-1RA treatment according to diverse areas of neuropsychological testing.

### 2.3. Study Selection

Duplicate articles were removed, and the title and abstract of all collected records were screened by 2 review authors (A.M.A. and G.M.) to identify articles that were potentially eligible for inclusion. Studies that clearly did not meet the inclusion criteria were excluded. Full texts of potentially relevant articles were retrieved and independently assessed for eligibility by 2 review team members (A.M.A. and G.M.). Disagreements were resolved through discussion and the reasons for exclusion were recorded.

Inclusion criteria were: (i) adults over 18 years of age; (ii) treatment with GLP-1RA; (iii) reporting information on cognitive performance tests evaluating different cognitive domains related to global cognitive functioning, attention, verbal, executive functioning, and memory; (iv) studies in humans; (v) written in English. Eligible GLP-1RA agents included: lixisenatide, liraglutide, semaglutide, albiglutide, dulaglutide, exenatide in the search. We included studies evaluating the use of GLP-1RA(s) independently of nutritional status, on cognitive performance evaluation tests following treatment. When differences in assessment arose between authors regarding study eligibility, a third author was consulted to review the differing opinions and make the final decision on study inclusion. Studies providing raw mean and standard deviation of cognitive tests for the outcome of interest were also included in the meta-analysis.

Exclusion criteria included: (i) non-original research (including reviews, comments, editorials, perspectives), (ii) non-English language because the review team did not have sufficient linguistic resources to reliably assess eligibility, methods and data extraction, (iii) retracted publications, (iv) animal or in vitro studies, (v) conference abstracts, (vi) lack of specific information for performing a meta-analysis.

### 2.4. Data Extraction and Outcomes

Data were abstracted independently by two researchers (F.D. and G.M.) using a standardized data collection form with a third independent investigator (A.M.A.) controlling the data collected. For every scientific paper, we abstracted the title, year of publication, authors, type of study design (open, placebo-controlled), intervention, numbers, cognitive performance tests and domains, and changes in cognitive test scores over time. We extracted data from the follow-up assessments at the time points reported in the included studies. When more than one assessment time point was reported, results at 18–24 months, or at the nearest time point available, were extracted. Included RCT protocols registered at ClincalTrial.gov website were searched for unreported data. The extracted data were cross-checked for inconsistencies and discrepancies, merged into a preliminary dataset, and independently reviewed by a third investigator (A.M.A.).

Given that specific areas of cognitive performance correspond to discrete areas of cerebral function, we investigated changes in neuropsychological measures of global cognitive functioning, attention, verbal, executive functioning, and memory. We evaluated performance scores in these five cognitive domains according to treatment or control groups. Cognitive function was assessed using the cognitive domains and instruments reported in the individual studies. No single cognitive domain, cognitive instrument, or assessment time point was prespecified as the primary cognitive outcome in the registered protocol. Therefore, the analyses of individual cognitive domains were considered exploratory. For global cognitive functioning, we evaluated the performance scores of the Mini-Mental State Examination (MMSE) or the Montreal Cognitive Assessment (MoCA). When more than one instrument assessing the same cognitive domain was reported within a study, a single instrument was selected to avoid double-counting correlated outcomes. For global cognition, the MMSE was preferentially selected when available; the MoCA was selected only when MMSE data were not reported. This selection rule was established post hoc after examination of the eligible studies and was therefore not prespecified in the original protocol. We included the Digit Symbol Substitution Test (DSST), the attention subdomain in the Repeatable Battery for the Assessment of Neuropsychological Status (RBANS) as measures of attention. Verbal domain tests included the Verbal Fluency Test. Trail making tests A and B were used to test for measures of executive functioning. Cognitive performance tests of memory included Digit Span Forward (DSF), delayed memory subdomain in the RBANS, and Spanish English Verbal Learning Test (SEVLT) (see Supplementary Table S1). Primary outcomes were comparative measures of cognitive performance between intervention and control groups.

### 2.5. Statistical Analysis

All statistical analyses were performed using Stata version 18 (StataCorp LLC, College Station, TX, USA). Cognitive domains were analyzed separately. To avoid unit-of-analysis errors, one cognitive outcome per trial within each domain was included in the quantitative synthesis. Continuous outcomes were summarized using Hedges’ g when compatible group-level means, participant-level standard deviations, and sample sizes were available. Model-based adjusted means and their confidence intervals or standard errors were not treated as raw means and participant-level standard deviations and not included in further analysis. For instruments in which lower scores indicate better performance, the direction of the effect was reversed so that positive Hedges’ g values consistently favored GLP-1RA treatment.

Random-effects models used restricted maximum likelihood (REML) estimation, with Knapp–Hartung inference when at least two compatible studies contributed to a pooled estimate. Between-study heterogeneity was described using tau^2^ and *I*^2^ and interpreted cautiously because of the small number of studies. No overall pooled estimate combining distinct cognitive domains was calculated. Formal funnel-plot asymmetry testing using Egger’s test was not performed because fewer than 10 independent studies contributed to each domain. Because one trial contributed 99.3% of the weight to the global cognition analysis, a sensitivity analysis excluding this dominant trial was also performed.

Statistical significance was set at a two-sided *p*-value < 0.05. Pooled results were expressed as Hedges’ g with 95% confidence intervals (95% CI) and displayed using forest plots. Domains represented by a single compatible study were reported as single-study effect estimates and were not interpreted as pooled meta-analytic effects. The dataset and statistical code used for the analyses are available from the corresponding author upon reasonable request.

### 2.6. Quality Assessment

The quality of each outcome of interest included was assessed independently by two authors (A.M.A. and G.M.) using the revised Cochrane risk-of-bias tool for randomized trials (RoB 2) [20]. The RoB 2 tool consists of five domains evaluating: (i) bias arising from the randomization process, (ii) bias due to deviations from intended interventions, (iii) bias due to missing outcome data, (iv) bias in outcome measurement, and (v) bias in selection of the reported results. Based on the individual domain risk-of-bias judgments, the overall risk-of-bias judgment is defined as “low risk of bias,” “some concerns,” or “high risk of bias”. As RoB 2 is outcome-specific, outcomes were initially considered at the outcome level; however, when multiple cognitive outcomes within the same trial shared the same methodological features and yielded identical domain-level judgments, they were presented jointly to avoid unnecessary duplication.

### 2.7. Certainty of Evidence Assessment

The certainty of evidence for each outcome of interest was evaluated using the Grading of Recommendations Assessment, Development and Evaluation (GRADE) approach [21]. GRADE assessments were performed both for outcomes included in quantitative meta-analyses and for outcomes represented by a single study. For the latter, the certainty assessment was based on the individual study estimate. As no specific cognitive domain, instrument, or follow-up time point was prespecified as the primary outcome of the review, domain-specific analyses were considered exploratory. As all included studies were randomized controlled trials, the certainty of evidence was initially rated as high and was subsequently downgraded by one or two levels when concerns were considered serious or very serious, respectively, across five domains: risk of bias, inconsistency, indirectness, imprecision, and publication bias. Risk of bias was informed by the RoB 2 assessments of the studies contributing to each outcome; for pooled estimates, their relative contribution to the meta-analysis was also considered. Inconsistency was assessed considering the magnitude and direction of individual study effects, overlap of confidence intervals, and statistical heterogeneity, including the *I*^2^ statistic. For outcomes represented by a single study, inconsistency was considered not assessable because no between-study comparison was possible and therefore did not result in downgrading. Indirectness was evaluated in relation to the prespecified review question, considering whether differences in study populations, GLP-1RA agents, comparators, cognitive instruments, and follow-up duration remained within the intended scope of the review. Imprecision was judged on the width of the 95% confidence interval around the pooled or individual study effect estimates and whether it included both no effect and clinically relevant benefit or harm. Publication bias was assessed qualitatively, considering the number and size of the available studies and the possibility of selective non-publication. For single-study outcomes, publication bias was evaluated qualitatively on the basis of the available evidence, acknowledging that formal assessment was not possible. The overall certainty of evidence was classified as high, moderate, low, or very low.

## 3. Results

**Figure 1:**
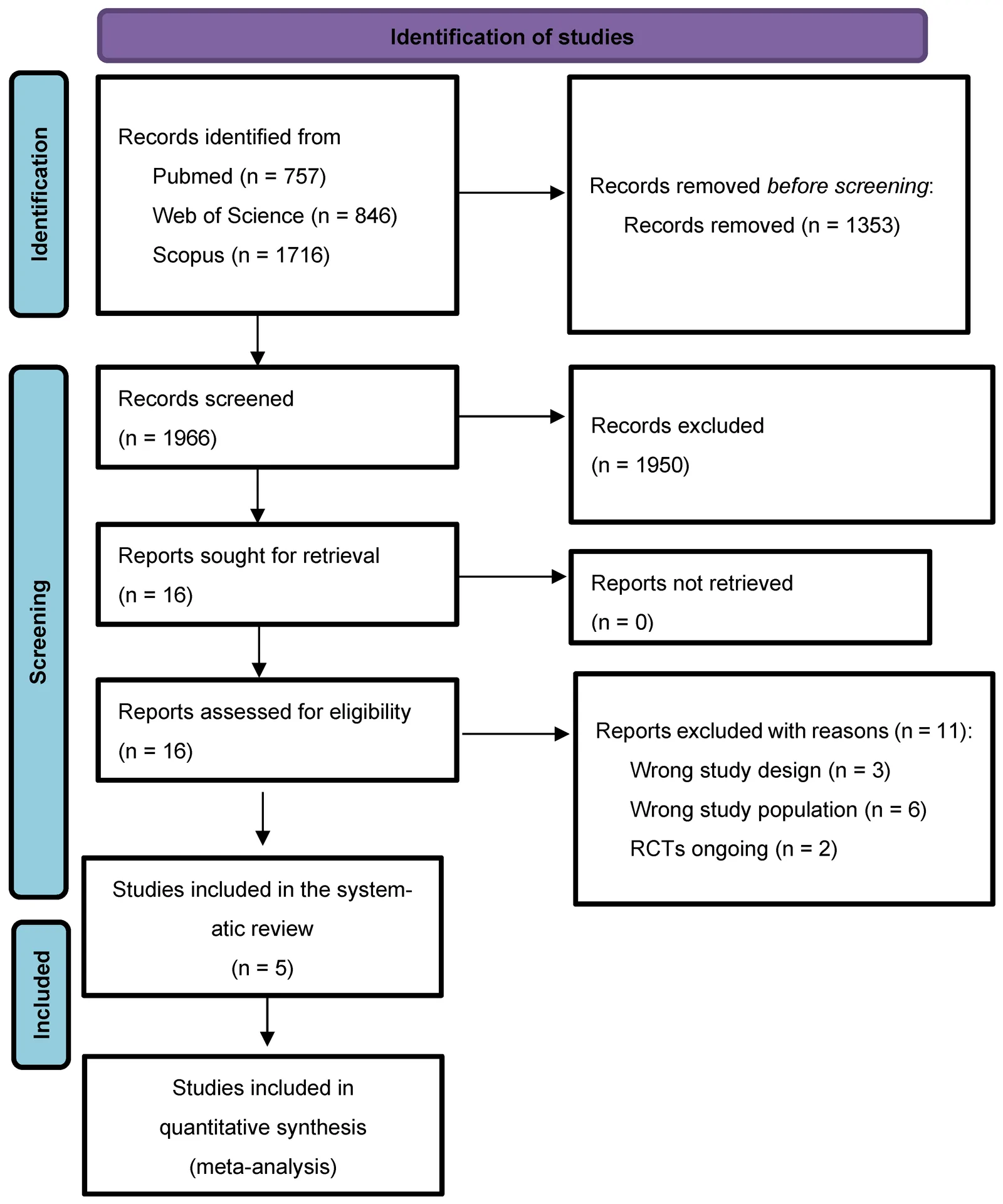
Prima flow diagram.

A literature search of three databases—PubMed, Web of Science and Scopus produced 3319 results, of which 1353 duplicates were removed, leaving 1966 records to be screened. Title and abstract screening excluded 1950 records. Full-text review excluded 11 articles. A total of five articles were included in the systematic review, and four were included in the quantitative synthesis. PRISMA flow chart is illustrated in Figure 1. Please see Supplementary Table S2, which provides detailed information on study selection and exclusion in the Supplementary Files.

Overall, five studies included data from different cognitive domains in 13,711 participants (Table 1). The mean duration follow-up was 28.4 months (range from 4 to 64). All eligible studies were RCTs comparing the use of GLP-1RA drugs (exenatide, dulaglutide, liraglutide) to a control group.

Of note, we reached out to the authors of the pilot study using exenatide [12] and to the authors of the GRADE trial [22], to ask for data regarding memory, verbal and executive functioning between groups; however, we did not receive any response regarding this information.

Participant baseline data, patient characteristics and neuropsychological tests analyzed in reports testing the use of GLP-1RA treatment are reported in Table 1. We found an overall mean age of 64.5 years. There were not any substantial differences according to age regarding intervention and comparison groups. Studies that included patients with MCI without T2DM used the GLP-1RA agent exenatide. The other two RCTS in those with Type 2 diabetes investigated the use of GLP-1RA agents, liraglutide and dulaglutide.

The study and instrument underlying each single-study estimate for delayed memory, executive function, and verbal function are reported in Table 2.

**Figure 2:**
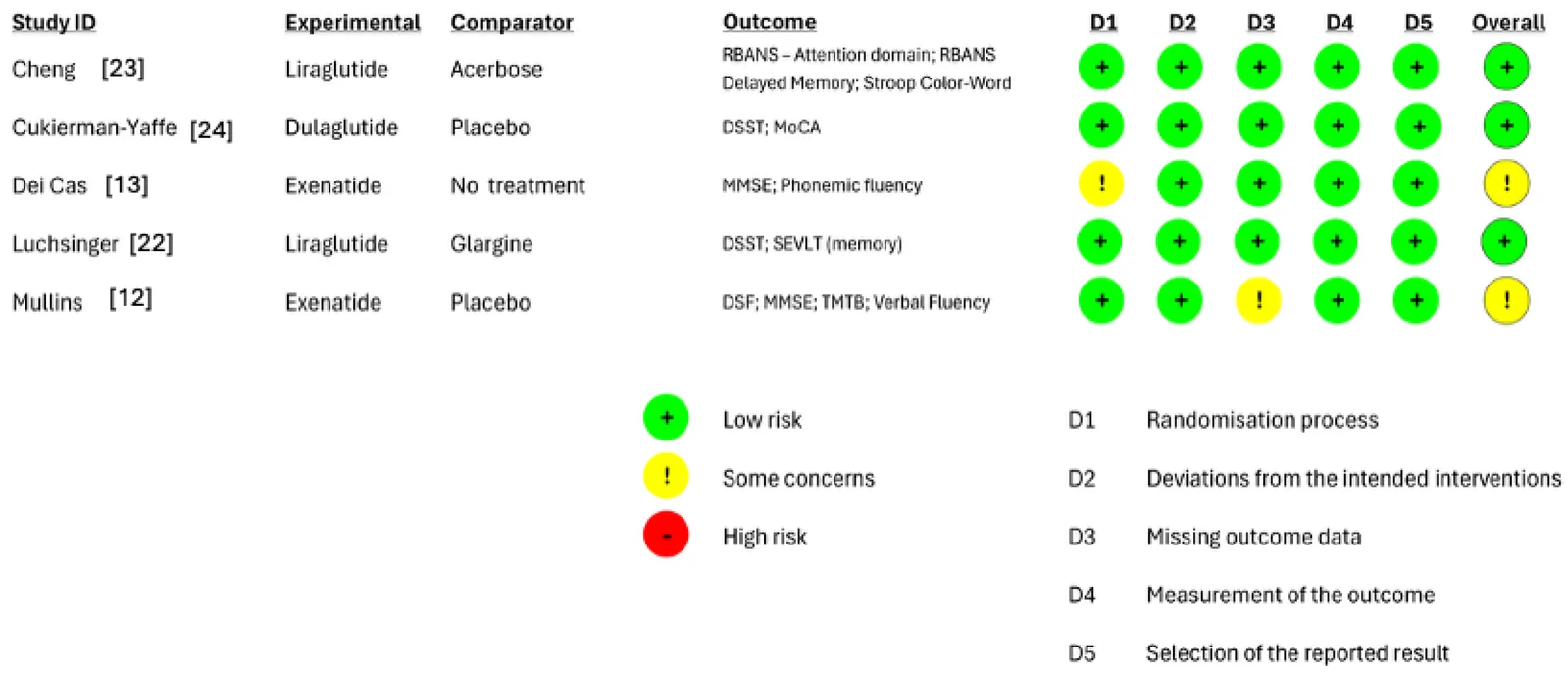
Risk of bias assessment summary for the included randomized controlled trials (RoB2) [12,13,22,23,24].

According to the outcome-specific RoB 2 assessment for the five trials included in the systematic review, outcomes from three trials [22,23,24] were judged at low risk of bias and two [12,13] raised some concerns (Figure 2). Specifically, insufficient reporting of the randomization procedures in Dei Cas et al. [13] raised some concerns regarding bias arising from the randomization process, whereas the proportion of randomized participants without baseline cognitive assessments in Mullins et al. [12] raised some concerns regarding bias due to missing outcome data. No included trial-outcome assessment was classified as high risk of bias in the revised analytical set.

**Figure 3:**
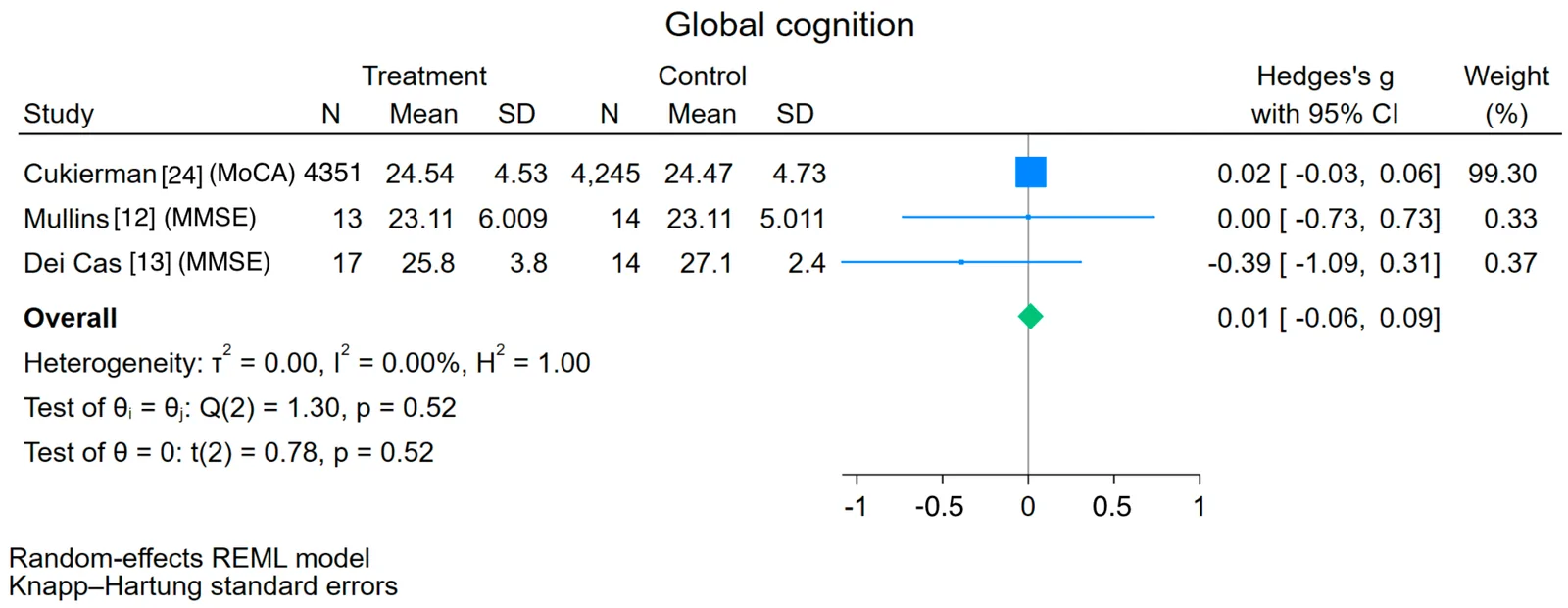
Global cognition: random-effects REML meta-analysis with Knapp–Hartung inference [12,13,24].

Following source-data audit, only analytically compatible continuous outcomes were used to calculate Hedges’ g, with one outcome per trial within each cognitive domain. Three studies contributed to the global cognition analysis [12,13,24]. The pooled random-effects estimate showed no evidence of a treatment effect (Hedges’ g = 0.014, 95% CI: −0.061 to 0.088; *p* = 0.515), with tau^2^ = 0 and *I*^2^ = 0%. However, the estimate was largely driven by the substantially larger Cukierman trial [24], which accounted for 99.3% of the meta-analytic weight (Figure 3).

**Figure 4:**
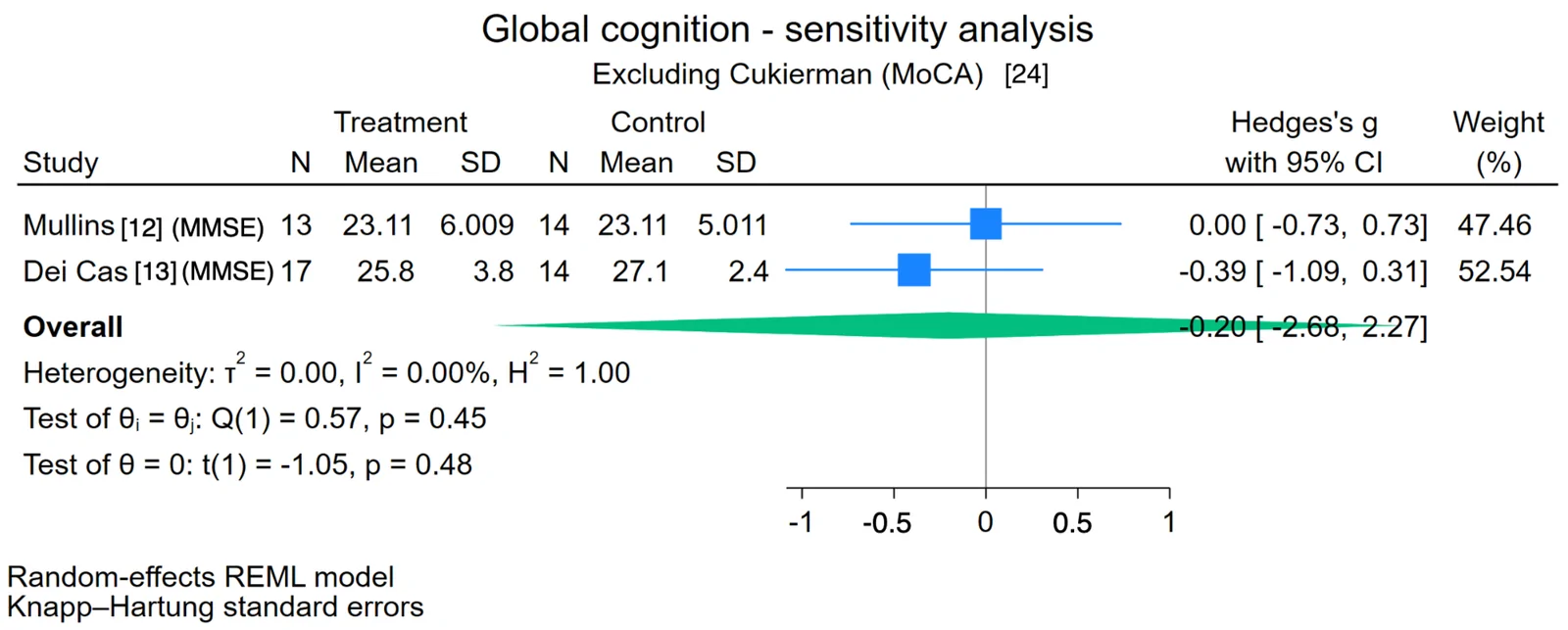
Sensitivity analysis for global cognition after exclusion of the dominant Cukierman (MoCA) trial [12,13].

In the sensitivity analysis excluding the dominant Cukierman trial [24] from global cognition, the remaining two studies yielded g = −0.205 (95% CI: −2.678 to 2.269; *p* = 0.484), with tau^2^ = 0 and *I*^2^ = 0%. This estimate was highly imprecise and did not provide evidence of either benefit or harm (Figure 4).

**Figure 5:**
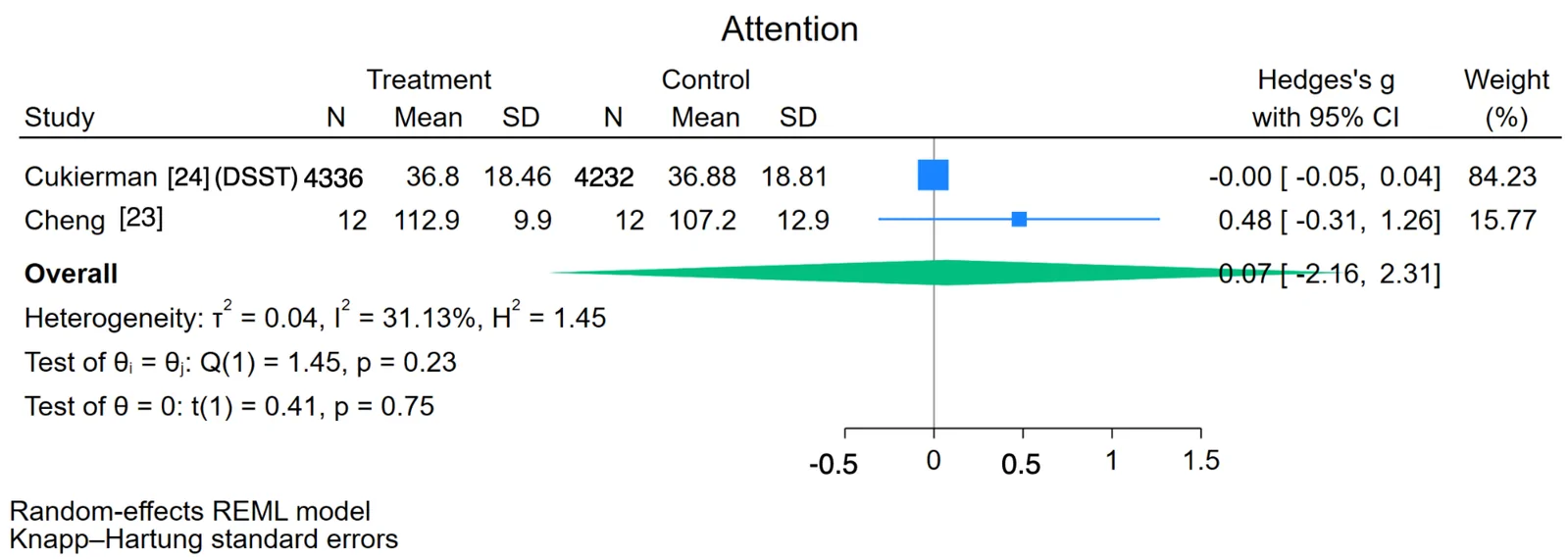
Attention: random-effects REML meta-analysis with Knapp–Hartung inference [23,24].

Two studies contributed to the attention domain [23,24]. The pooled estimate was g = 0.072 (95% CI: −2.164 to 2.308; *p* = 0.753), with tau^2^ = 0.036 and *I*^2^ = 31.1%. The very wide Knapp–Hartung confidence interval reflects the substantial uncertainty associated with pooling only two studies (Figure 5).

For memory, executive function, and verbal function, only one compatible study was available per domain; these findings were therefore reported as single-study effect estimates rather than pooled meta-analytic effects [12,13,23]. The corresponding estimates were g = 1.10 (0.27 to 1.93) for delayed memory, g = 0.611 (95% CI: −0.181 to 1.403) for executive function, and g = −0.554 (95% CI: −1.257 to 0.148) for verbal function. No pooled forest plot was generated for domains represented by a single study.

GRADE assessment, reported in Table 3, indicated high-certainty evidence for global cognition, suggesting little to no effect of treatment on this outcome. No downgrade for indirectness was applied because differences in study population, GLP-1RA agent, comparator, cognitive instrument, and follow-up remained within the prespecified scope of the review. Also, although Cukierman-Yaffe et al. contributed approximately 99% of the meta-analytic weight, this was not considered a source of indirectness because the trial population, intervention, comparator, and outcome were directly relevant to the prespecified review question. No downgrade for risk of bias was applied because Cukierman-Yaffe et al., which contributed almost all the statistical weight, was judged at low risk of bias, while the concerns identified in the two smaller studies were considered unlikely to materially affect the pooled estimate. No downgrade for inconsistency was applied because the individual study effects showed no important discordance and statistical heterogeneity was negligible (*I*^2^ = 0%). No downgrade for imprecision was applied because the pooled estimate was close to the null and the 95% confidence interval was narrow (Hedges’ g 0.01, 95% CI: −0.06 to 0.09), excluding effects of a magnitude considered clinically important. Finally, although formal assessment of publication bias was limited by the small number of studies, we found no specific indication warranting downgrading. In contrast, the certainty of evidence for attention was rated as low because of very serious imprecision, indicating substantial uncertainty regarding the direction and magnitude of the effect. Among single-study findings, certainty of evidence was rated as low for executive function due to very serious imprecision, moderate for memory due to serious imprecision, and low for verbal fluency due to serious risk-of-bias concerns and serious imprecision. As these outcomes were represented by a single trial, inconsistency was not assessable and did not result in downgrading.

## 4. Discussion

This systematic review and meta-analysis evaluated the effects of GLP-1RA treatment on cognitive performance in adults at risk of AD, specifically with Type 2 diabetes or without Type 2 diabetes (MCI). We found no evidence supporting GLP-1RA(s) on cognitive performance for adults at risk of AD. Global cognition showed no evidence of a treatment effect, whereas the attention estimate remained not significant even following sensitivity analysis. Cognitive performance domains evaluating memory, executive function, and verbal function were each represented by a single compatible study and therefore, could not be interpreted as pooled domain-specific treatment effects.

General cognitive assessments with MMSE or MoCA lack sensitivity to modest domain-specific changes, particularly in the early stages of cognitive decline [25]. Because in-depth neuropsychological testing of specific domains, including attention, verbal abilities, memory, and executive functioning, can reflect the integrity of areas of cerebral functioning, we specifically examined the available literature of RCTs on these individual outcomes. Clinically, subtle impairment of one or more of these domains often occurs before they can be detected using the MMSE or MoCA [25]. It is important to note that domain-specific analyses of cognitive outcomes are gaining increasing attention in the literature, as illustrated by the recent meta-analysis by Xu et al. [26], which similarly evaluated distinct cognitive domains separately rather than combining heterogeneous neuropsychological outcomes into a single overall estimate. However, this meta-analysis [26] was designed to specifically evaluate cognitive performance in patients with diagnosed neurodegenerative diseases, namely AD or Parkinson’s disease, as well as in patients with Type 2 Diabetes, following GLP-1RA intervention. These authors also included a paper by Li et al. [27] in their analyses, whereas we did not include it in the present meta-analysis because it was not considered an RCT, as stated by the authors in the original article.

Chronic hyperglycemia has been consistently associated with an increased risk of cognitive impairment and suboptimal glycemic control may be associated with modest decrements of cognitive performance [28]. Although both Type 2 diabetes trials included in our paper showed comparable improvements in glycemic control over time in both the GLP-1RA and control groups, participants receiving GLP-1RA in the paper by Cheng et al. were the only group with available data highlighting improvements in delayed memory, attention and executive function, while no outcomes were found for global cognition even after adjustment for glycemic control [23]. Another report by Luchsinger et al. [22] did not find any significant associations between time-weighted HbA1c and cognitive outcomes over a 4.3-year follow-up period. Unfortunately, we were unable to compare these studies due to the lack of data regarding cognitive test scores.

It is widely recognized that determinants of low-grade inflammation, including overnutrition, obesity, and aging, contribute to increased cardiovascular risk and cognitive decline [5,6,29]. Given that GLP-1RA interventions reduce body weight and improve metabolic pathways involved in glucose homeostasis, oxidative stress, and inflammation, GLP-1RA(s) may improve cardiovascular risk profiles, especially in those overweight and/or with T2DM [30]. In fact, the trial by Cukierman-Yaffe et al. [24] specifically included participants with a history of cardiovascular disease. Even though the results from the sensitivity analysis for global cognition were performed after excluding the study by Cukierman-Yaffe, we did not find any significant treatment effect change (Figure 4).

GLP-1 receptors are highly concentrated in the hypothalamus, hippocampus, and cerebral cortex [31,32]. GLP-1RA(s) can cross the blood–brain barrier and preclinical studies have suggested neuroprotective mechanisms, including effects on amyloid-related pathways, tau phosphorylation, oxidative stress, synaptic function, and neuronal plasticity [7,33,34]. These biological observations provide mechanistic plausibility but should not be interpreted as evidence of a clinically meaningful cognitive benefit.

In the revised quantitative analysis, the favorable delayed-memory estimate derives from a single small trial [23] and therefore requires confirmation in larger, specifically designed randomized trials. The revised domain-specific analysis does not support a consistent treatment effect across cognitive domains. Attention was pooled from only two compatible studies and had a very wide confidence interval, while executive and verbal outcomes were represented by single studies. Individual trials have reported signals in selected cognitive measures at follow-up times, but these findings remain heterogeneous and should be interpreted descriptively rather than as evidence of a reproducible domain-specific benefit.

As previously mentioned, Type 2 diabetes is associated with an increased risk of AD and is frequently observed in individuals who progress from MCI to AD. However, we identified and included only two small RCTs in MCI in our analysis. Mullins et al. [12] evaluated exenatide administered at 5 mcg twice daily, titrated to 10 mcg twice daily, over 18 months, while Dei Cas et al. [13] investigated a long-acting formulation of exenatide at 2 mg once weekly for 8 months. Given heterogeneity in study populations, pharmacological formulations, dosing regimens, and intervention periods, current evidence remains insufficient to draw substantiated conclusions about the efficacy of GLP-1RA(s) in patients with MCI. Future trials are needed to test a potential role of GLP-1RA(s) on specific cognitive domains in MCI with and without Type 2 Diabetes.

In this paper, we specifically aimed to investigate cognitive domain outcomes among individuals at risk of AD. Given the potential of GLP-1RA(s) to improve cognitive performance, as suggested by previous preclinical and epidemiological evidence [9,10], future RCTs specifically designed to assess their effects on different cognitive domains in well-defined at-risk populations may be warranted.

Unfortunately, different RCTs testing the impact of GLP-1RA use on improving cognitive abilities or stabilizing cognitive performance in persons with early AD have not shown positive findings [35]. A recent report regarding findings from two large multicenter and randomized trials, the Evoke and Evoke+ trials, included adults aged between 55 and 85 years with mild dementia due to an AD diagnosis (semaglutide, n = 928; placebo, n = 927; and semaglutide, n = 976; placebo, n = 977, respectively) [35]. Both trials tested once daily oral semaglutide 14 mg or placebo for 156 weeks [35]. The authors did not find that semaglutide was efficacious in slowing clinical progression, as measured by cognitive and functional changes on the Clinical Dementia Rating—sum of boxes (CDR-SB) in early AD. The authors found no differences in global cognition based on MoCA or MMSE results, which also confirms our findings from previous trials in those at risk of AD. This may be explained by the fact that neurodegeneration process has already begun, and the potential neuroprotective effect of GLP-1RA(s) is most likely limited due to the presence of neuronal loss and synaptic degeneration [36,37]. Indeed, GLP-1RA intervention might have potential for those individuals at risk of AD who are asymptomatic. Considering the overall safety profile, specifically designed RCTs may be warranted.

Another recent multicenter RCT, the ELAD study, evaluated the use of liraglutide in adults with mild to moderate AD with no diabetes and did not find any significant differences in cerebral glucose metabolism after 12 months; however, they found a slight improvement in executive function tests [38].

The principal limitations are the small number of analytically compatible trials within each cognitive domain, differences in study populations, GLP-1RA agents, comparators, follow-up duration, and neuropsychological instruments, and the disproportionate influence of one large trial on global cognition. With only two or three studies in pooled analyses, estimates of between-study heterogeneity are imprecise; therefore, tau^2^ and *I*^2^ should be interpreted cautiously. Formal funnel-plot asymmetry testing was not performed because fewer than 10 independent studies contributed to each domain. The sensitivity analysis excluding the dominant Cukierman trial [24] produced a highly imprecise estimate and should not be interpreted as confirming robustness. An additional limitation is that no single cognitive domain, instrument, or assessment time point was prespecified as the primary cognitive outcome in the registered protocol. Accordingly, the domain-specific analyses should be regarded as exploratory and may be subject to multiplicity and selective outcome reporting. These findings should therefore be interpreted cautiously and considered hypothesis-generating.

## 5. Conclusions

Current randomized evidence is insufficient to establish a consistent beneficial effect of GLP-1RA(s) on cognitive function. Global cognition showed no evidence of a treatment effect, while the pooled attention estimate was highly imprecise. Favorable findings for delayed memory and executive function arose from a single small, randomized trial [23] and should therefore be considered exploratory and hypothesis-generating. The evidence is limited by the small number of analytically compatible trials, heterogeneous populations, interventions and cognitive measures, and risk-of-bias concerns. Larger randomized controlled trials specifically designed to assess cognition, with standardized and prespecified cognitive outcomes and adequate follow-up, are required to determine whether GLP-1RA(s) hold potential in adults at risk of developing AD.

## Figures and Tables

**Table 1:** Characteristics of the studies included in the systematic review.

Author (Year)	Study Duration (Months)	Total Sample Size (n)	Inclusion Criteria	Age, Mean (yr)		GLP-1RA Drug	Control	Neuropsychological Test Domains Included in the Systematic Review (Sample Size)
				Intervention	Comparison			
Mullins RJ et al. (2019) [12]	18	27	Age ≥ 60 MCI No DM	71.7	74.0	Exenatide	Placebo	Global cognition: MMSE (27) Executive: TMT (27) * Memory: DSF (27) * Verbal: verbal fluency (27) *
Dei Cas et al. (2024) [13]	8	31	Age ≥ 50 to ≤80 yrs MCI No DM	74	72	Exenatide	No treatment	Global cognition: MMSE (31) Verbal: phonemic fluency (31)
Luchsinger JA et al. (2025) [22]	48	3721	Age ≥ 30 years T2DM In treatment with Metformin	57.3	56.9	Liraglutide	Other anti-diabetic agent	Attention: DSST * Memory: SEVLT *
Cheng H et al. (2022) [23]	4	36	T2DM HbA1c ≥ 7.0% In treatment with metformin	51.9	56.4	Liraglutide	Other anti-diabetic agent	Attention: RBANS (24) Executive: Stroop Color-Word Test (24) Memory: RBANS (24)
Cukierman-Yaffe T et al. (2020) [24]	64	9901	Age ≥ 50 T2DM BMI ≥ 23	65.5	65.5	Dulaglutide	Placebo	Attention: DSST (8568) Global cognition: MoCA (8597)

* Outcome not included in the statistical analysis. Abbreviations: T2DM = Type 2 Diabetes; MCI = Mild Cognitive Impairment; MMSE = Mini-Mental State Examination; TMT = Trail Making Test; DSF = Digit Span Forward; MoCA = Montreal Cognitive Assessment; DSST = Digit Symbol Substitution Test; SEVLT = Spanish English Verbal Learning Test; and RBANS = Repeatable Battery for the Assessment of Neuropsychological Status.

**Table 2:** Outcome-level characteristics and rationale for selection of cognitive outcomes included in the quantitative analyses.

Cognitive Domain	Study	Instrument	Follow-Up Used	Analytical Sample Size, GLP-1RA/Control	Type of Estimate Used	Rationale for Outcome Selection
Global cognition	Cukierman-Yaffe et al. [24]	Montreal Cognitive Assessment (MoCA)	24 months	4351/4245	Intermediate post-intervention values; Hedges’ g calculated from arm-level means and SDs	MoCA directly assesses global cognitive function. The 24-month scheduled assessment provided the time point most in line with the study final time point.
Global cognition	Dei Cas et al. [13]	Mini-Mental State Examination (MMSE)	8 months	17/14	Final post-intervention values; Hedges’ g calculated from arm-level means and SDs	MMSE directly assesses global cognitive function.
Global cognition	Mullins et al. [12]	Mini-Mental State Examination (MMSE)	18 months	13/14	Final post-intervention values; Hedges’ g calculated from arm-level means and SDs	MMSE was selected as the measure of global cognition.
Attention	Cukierman-Yaffe et al. [24]	Digit Symbol Substitution Test (DSST)	24 months	4336/4232	Intermediate post-intervention values; Hedges’ g calculated from arm-level means and SDs	DSST assesses the attention/processing-speed domain. The 24-month scheduled assessment provided the time point most in line with other studies’ final time points.
Attention	Cheng et al. [23]	RBANS Attention Index	4 months	12/12	Final post-intervention values; Hedges’ g calculated from arm-level means and SDs	The RBANS Attention Index directly assesses the cognitive domain of interest.

**Table 3:** Summary of pooled effects and GRADE certainty of evidence for cognitive outcomes.

Outcome	Studies	Participants	Hedges’ g (95% CI)	Certainty (GRADE)	Reasons for Downgrading
Attention	2	8592	0.07 (−2.16 to 2.31)	⊕⊕◯◯ Low	Very serious imprecision
Executive function	1	24	0.61 (−0.18 to 1.40)	⊕⊕◯◯ Low	Very serious imprecision
Global cognition	3	8654	0.01 (−0.06 to 0.09)	⊕⊕⊕⊕ High	None
Memory	1	24	1.10 (0.27 to 1.93)	⊕⊕⊕◯ Moderate	Serious imprecision
Verbal fluency	1	31	−0.55 (−1.26 to 0.15)	⊕⊕◯◯ Low	Serious risk of bias; serious imprecision

For cognitive domains represented by a single trial, the reported effect corresponds to the individual study estimate rather than to a pooled meta-analytic estimate. No downgrade for inconsistency is applied when only one study contributes to the outcome.

## Data Availability

No new data were created or analyzed in this study. Data sharing is not applicable to this article.

## Supplementary Materials

The following supporting information can be downloaded at: https://www.mdpi.com/article/10.3390/nu18203312/s1: File S1: Search strategy; Table S1: Cognitive domains and correlated neuropsychological testing; Table S2: Studies excluded with reasons as reported in the PRISMA flow diagram. Table S3: Outcome-level characteristics and rationale for selection of cognitive outcomes included in the quantitative analyses.
