# Supplementary material for: Impact of GLP-1 Receptor Agonists on Cognitive Performance in Adults at Risk of Alzheimer’s Disease: A Systematic Review and Meta-Analysis of Randomized Controlled Trials
Source: Nutrients. 2026 Oct 9;18(20):3312. doi: 10.3390/nu18203312 (PMC13708722; doi:10.3390/nu18203312)
Supplement: Supplementary file 1 [file nutrients-18-03312-s001.zip › nutrients-4537403-supplementary.pdf]

Supplementary file

File S1. Search strategy

Pubmed

("glucagon-like peptide-1 receptor agonists"[Mesh] OR "glucagon-like peptide-1 receptor agonist*"[tiab] OR "lixisenatide" [supplementary concept] OR "liraglutide"[Mesh] OR "liraglutide"[tiab] OR "lixisenatide"[tiab] OR "semaglutide"[Mesh] OR “semaglutide” [tiab] OR “albiglutide” [tiab] OR "dulaglutide" [supplementary concept] OR "dulaglutide"[tiab] OR "exenatide"[Mesh] OR "exenatide"[tiab] OR "ac3174" [supplementary concept] OR "ac3174" [tiab] OR “glp-1 agonist*”[tiab] OR “glp-1ra*”[tiab] OR “glp-1 analog*”[tiab] OR “incretin mimetic*”[tiab])

AND

("Dementia*"[tiab] OR "Cognitive impairment*"[tiab] OR "MCI"[tiab] OR "Neurocognitive disorder*"[tiab] OR "Cognitive decline"[tiab] OR "Memory disorder*"[tiab] OR "Memory impairment"[tiab] OR "Cognitive dysfunction*"[tiab] OR "Brain degeneration*"[tiab] OR "Mental deterioration*"[tiab] OR "Alzheimer*"[tiab] OR "Lewy bod*"[tiab] OR "DLB"[tiab] OR "Parkinson*"[tiab] OR "PDD"[tiab] OR "PD-D"[tiab] OR "VAD"[tiab] OR "FTD"[tiab] OR "Primary progressive aphasia*"[tiab] OR "Normal pressure hydrocephalus*"[tiab] OR "Creutzfeldt-Jakob"[tiab] OR "Wernicke-Korsakoff"[tiab] OR "WKS"[tiab] OR "Huntington*"[tiab] OR "Posterior cortical atrophy"[tiab] OR "MMSE"[tiab] OR "Mini Mental State Examination"[tiab] OR "neuropsychological test battery"[tiab] OR "NTB"[tiab] OR "neurobehavioral test battery"[tiab] OR "neuropsychological evaluation"[tiab] OR "neuropsychological assessment"[tiab] OR "GP-COG"[tiab] OR "assessment of cognition"[tiab] OR "evaluation of cognition"[tiab] OR "cognitive assessment"[tiab] OR "cognitive evaluation"[tiab] OR "cognitive test*"[tiab] OR "short test* of mental status"[tiab] OR "STMS"[tiab] OR "short portable mental status questionnaire*"[tiab] OR "SPMSQ"[tiab] OR "Pfeiffer test"[tiab] OR "clock drawing test"[tiab] OR "CDT"[tiab] OR "MDB"[tiab] OR "mental deterioration battery"[tiab] OR "FAB"[tiab] OR "frontal assessment battery"[tiab] OR "ADAS-Cog*"[tiab] OR "AD assessment scale-cognition*"[tiab] OR "Digit span"[tiab] OR "NCSD"[tiab] OR "BPSD"[tiab] OR "global deterioration scale"[tiab] OR "neuropsychological tests"[MeSH] OR "dementia"[MeSH] OR "cognitive dysfunction"[MeSH])

Scopus

TITLE-ABS-KEY ("glucagon-like peptide-1 receptor agonist*" OR "liraglutide" OR "lixisenatide" OR "semaglutide" OR “semaglutide” OR “albiglutide” OR "dulaglutide" OR “exenatide" OR "ac3174” OR “glp-1 agonist*” OR “glp-1ra*” OR “glp-1 analog*” OR “incretin mimetic*”)

AND

TITLE-ABS-KEY ("Dementia*" OR "Cognitive impairment*" OR "MCI" OR "Neurocognitive disorder*" OR "Cognitive decline" OR "Memory disorder*" OR "Memory impairment" OR "Cognitive dysfunction*" OR "Brain degeneration*" OR "Mental deterioration*" OR "Alzheimer*" OR "Lewy bod*" OR "DLB" OR "Parkinson*" OR "PDD" OR "PD-D" OR "VAD" OR "FTD" OR "Primary progressive aphasia*" OR "Normal pressure hydrocephalus*" OR "Creutzfeldt-Jakob" OR "Wernicke-Korsakoff" OR "WKS" OR "Huntington*" OR "Posterior cortical atrophy" OR "MMSE" OR "Mini Mental State Examination" OR "neuropsychological test battery" OR "NTB" OR "neurobehavioral test battery" OR "neuropsychological evaluation" OR "neuropsychological assessment" OR "GP-COG" OR "assessment of cognition" OR "evaluation of cognition" OR "cognitive assessment" OR "cognitive evaluation" OR "cognitive test*" OR "short test* of mental status" OR "STMS" OR "short portable mental status questionnaire*" OR "SPMSQ" OR "Pfeiffer test" OR "clock drawing test" OR "CDT" OR

"MDB" OR "mental deterioration battery" OR "FAB" OR "frontal assessment battery" OR "ADAS-Cog*" OR "AD assessment scale-cognition*" OR "Digit span" OR "NCSD" OR "BPSD" OR "global deterioration scale")

Web of Science

TS=("glucagon-like peptide-1 receptor agonist*" OR "liraglutide" OR "lixisenatide" OR "semaglutide" OR “semaglutide” OR “albiglutide” OR "dulaglutide" OR “exenatide" OR "ac3174” OR “glp-1 agonist*” OR “glp-1ra*” OR “glp-1 analog*” OR “incretin mimetic*”)

AND

TS= ("Dementia*" OR "Cognitive impairment*" OR "MCI" OR "Neurocognitive disorder*" OR "Cognitive decline" OR "Memory disorder*" OR "Memory impairment" OR "Cognitive dysfunction*" OR "Brain degeneration*" OR "Mental deterioration*" OR "Alzheimer*" OR "Lewy bod*" OR "DLB" OR "Parkinson*" OR "PDD" OR "PD-D" OR "VAD" OR "FTD" OR "Primary progressive aphasia*" OR "Normal pressure hydrocephalus*" OR "Creutzfeldt-Jakob" OR "Wernicke-Korsakoff" OR "WKS" OR "Huntington*" OR "Posterior cortical atrophy" OR "MMSE" OR "Mini Mental State Examination" OR "neuropsychological test battery" OR "NTB" OR "neurobehavioral test battery" OR "neuropsychological evaluation" OR "neuropsychological assessment" OR "GP-COG" OR "assessment of cognition" OR "evaluation of cognition" OR "cognitive assessment" OR "cognitive evaluation" OR "cognitive test*" OR "short test* of mental status" OR "STMS" OR "short portable mental status questionnaire*" OR "SPMSQ" OR "Pfeiffer test" OR "clock drawing test" OR "CDT" OR "MDB" OR "mental deterioration battery" OR "FAB" OR "frontal assessment battery" OR "ADAS-Cog*" OR "AD assessment scale-cognition*" OR "Digit span" OR "NCSD" OR "BPSD" OR "global deterioration scale")

Table S1. Cognitive domains and correlated neuropsychological testing

Cognitive domain	*Neuropsychological test*	*Abbreviation*	*Evaluation*	*Reference*
Global cognitive function	Mini-Mental State Examination	MMSE	Global cognitive function (orientation, memory, attention, language, and visuospatial skills)	Folstein et al., 1975 [39]
	Montreal Cognitive Assessment	MoCA	Global cognitive function (memory, visuospatial abilities, executive functions, attention/concentration, language, conceptual thinking, and orientation)	Nasreddine et al., 2005 [40]
Attention	Digit Symbol Substitution Test	DSST	Processing speed, complex attention, visual scanning, working memory	Wechsler 1981 [41]
	Repeatable Battery for the Assessment of Neuropsychological Status – attention domain	RBANS	Immediate Memory, Delayed Memory, Attention, Language, and Visuospatial/Constructional abilities. Only attention domain was considered for the analysis	Randolph et al., 1998 [42]
Verbal function	Verbal fluency	•	Semantic and Phonemic fluency	Lezak, 2012 [43]
Executive function	Trail Making test A	TMT-A	Processing speed, visual scanning, visuomotor tracking	Reitan 1955 [44]
	Trail Making Test B	TMT-B	cognitive flexibility, executive function, and task-switching abilities	Reitan 1955 [44]
Memory	Digit Span Forward	DSF	Short term memory	Wechsler 1981 [41]
	Repeatable Battery for the Assessment of Neuropsychological Status – memory domain	RBANS-delayed memory	Delayed memory recall	Randolph et al., 1998 [42]
	Spanish English Verbal Learning Test	SEVLT – delayed memory	Delayed memory recall	González HM et al. 2001 [45]

Table S2. Studies excluded with reasons as reported in the PRISMA flow diagram

**Study evaluated**	**Reason for exclusion**
Ateh O et al. [46]	Wrong study population - HIV + participants only
Cozza A et al. [47]	Wrong study design - Review analysis not RCT
Cummings JL et al. [48]	RCT ongoing at time of analysis – potential wrong population – participants already had a diagnosed AD

Daubitz T. [49]	Wrong study population — participants had early Parkinson’s disease rather than MCI/eligible cognitive impairment.
De Giorgi R et al. [50]	Wrong study design - observational cohort study evaluating antidiabetic treatment patterns; no eligible GLP-1RA-related cognitive outcome data.
Edison P et al. [51]	RCT was ongoing at time of analysis with potential wrong study population – participants with established AD
Kimura Y et al. [52]	Wrong study population – participants with Parkinson’s disease; study protocol with no eligible outcome data.
Kjærgaard, K. et al. [53]	Wrong study population – preclinical animal study.
Li Q et al. [54]	Wrong study design – prospective open-label non-randomized study.
Perna S et al. [55]	Wrong study population - the incretin group included both GLP-1RA and DPP-4 inhibitors.
Vadini F et al. [56]	Wrong study population - No follow-up cognitive data specific to participants with type 2 diabetes mellitus.

39. Folstein MF, Folstein SE, McHugh PR. "Mini-mental state". A practical method for grading the cognitive state of patients for the clinician. J Psychiatr Res. 1975 Nov;12(3):189-98. doi: 10.1016/0022-3956(75)90026-6. PMID: 1202204
40. Nasreddine ZS, Phillips NA, Bédirian V, Charbonneau S, Whitehead V, Collin I, Cummings JL, Chertkow H. The Montreal Cognitive Assessment, MoCA: a brief screening tool for mild cognitive impairment. J Am Geriatr Soc. 2005 Apr;53(4):695-9. doi: 10.1111/j.1532-5415.2005.53221.
41. Wechsler D. WAIS-R manual: Wechsler Adult Intelligence Scale–Revised. New York: Psychological Corporation; 1981.
42. Randolph C, Tierney MC, Mohr E, Chase TN. The Repeatable Battery for the Assessment of Neuropsychological Status (RBANS): preliminary clinical validity. J Clin Exp Neuropsychol. 1998 Jun;20(3):310-9. doi: 10.1076/jcen.20.3.310.823. PMID: 9845158.
43. Lezak MD, Howieson DB, Bigler ED, Tranel D. *Neuropsychological assessment.* 5th ed. New York: Oxford University Press; 2012.
44. 44. Reitan RM. The relation of the trail making test to organic brain damage. J Consult Psychol. 1955 Oct;19(5):393-4. doi: 10.1037/h0044509. PMID: 13263471.
45. 45. González HM, Mungas D, Reed BR, Marshall S, Haan MN. A new verbal learning and memory test for English- and Spanish-speaking older people. J Int Neuropsychol Soc. 2001 Jul;7(5):544-55. doi: 10.1017/s1355617701755026. PMID: 11459106.
46. Atieh O, Daher J, Abboud M, Wu Q, Sattar A, Baissary J, Koberssy Z, Labbato D, Eckard AR, McComsey GA. Effects of Semaglutide on Cognitive Function in People With HIV: A Randomized, Controlled Trial. Clin Infect Dis. 2026 May 20;82(5):753-761. doi: 10.1093/cid/ciaf577. PMID: 41098140; PMCID: PMC13189676.
47. Cozza A, Chinigò C, Filicetti E, Greco GI, Lappano R, Marinaro C, Muglia L, Soraci L, Corsonello A, Lattanzio F, Volpentesta M. Effects of antidiabetic medications on the relationship between type 2 diabetes mellitus and cognitive impairment. Ageing Res Rev. 2025 Dec;112:102834. doi: 10.1016/j.arr.2025.102834. Epub 2025 Jul 12. PMID: 40659290.
48. Cummings JL, Atri A, Feldman HH, Hansson O, Sano M, Knop FK, Johannsen P, León T, Scheltens P. evoke and evoke+: design of two large-scale, double-blind, placebo-controlled, phase 3 studies evaluating efficacy, safety, and tolerability of semaglutide in early-stage symptomatic Alzheimer's disease. Alzheimers Res Ther. 2025 Jan 8;17(1):14. doi: 10.1186/s13195-024-01666-7. PMID: 39780249; PMCID: PMC11708093.
49. Daubitz T. Lixisenatid bremst Parkinson. Deutsche Apotheker Zeitung. 2024;20:15 May 2024
50. De Giorgi R, Koychev I, Adler AI, Cowen PJ, Harmer CJ, Harrison PJ, Taquet M. 12-month neurological and psychiatric outcomes of semaglutide use for type 2 diabetes: a propensity-score matched cohort study. EClinicalMedicine. 2024 Jul 10;74:102726. doi: 10.1016/j.eclinm.2024.102726. PMID: 39764175; PMCID: PMC11701436.
51. Edison P, Femminella GD, Ritchie C, Nowell J, Holmes C, Walker Z, Ridha B, Raza S, Livingston NR, Frangou E, Love S, Williams G, Lawrence R, Mcfarlane B, Archer H, Coulthard E, Underwood BR, Koranteng P, Karim S, Bannister C, Perneczky R, Prasanna A, Junaid K, McGuinness B, Nilforooshan R, Macharouthu A, Donaldson A, Thacker S, Russell G, Malik N, Mate V, Knight L, Kshemendran S, Holscher C, Mansouri A, Chester-Jones M, Holmes J, Tan T, Williams S, Ashraf A, Brooks DJ, Harrison J, Hinz R, Tadros G, Passmore AP, Ballard C. Liraglutide in mild to moderate Alzheimer's disease: a phase 2b clinical trial. Nat Med. 2026 Jan;32(1):353-361. doi: 10.1038/s41591-025-04106-7. Epub 2025 Dec 1. PMID: 41326666; PMCID: PMC12823385.
52. Kimura Y, Koda T, Kurakami H, Sakamoto S, Iwasaki K, Asai K, Ge L, Kato H, Tsuboi T, Matsukawa N, Kano O, Matsuse D, Tomiyama M, Yokoe M, Nagai Y, Mochizuki H. Disease-modifying effect, safety and optimal dose of oral semaglutide tablets for patients with Parkinson's disease (MOST-ABLE study): protocol for a randomised, double-blind, placebo-controlled study. BMJ Open. 2025 Dec 24;15(12):e112318. doi: 10.1136/bmjopen-2025-112318. PMID: 41448692; PMCID: PMC12742159.
53. Kjærgaard, K., Daugaard Mikkelsen, A. C., Bay-Richter, C., Landau, A. M., Eriksen, P. L., Elfving, B., Tamnaloo, F., Yeoman, J. R., Dutoit-Hamilton, S., Rose, C. F., Vilstrup, H., Mookerjee, R. P., & Thomsen, K. L. (2023). P42 Semaglutide Reduces Cognitive Dysfunction in Experimental Non-Alcoholic

Steatohepatitis Through Anti-Inflammatory and Neuroprotective Effects. American Journal of Gastroenterology, 118(9S), S28-S29. https://doi.org/10.14309/01.ajg.0000948452.01988.0c
54. Li Q, Jia M, Yan Z, Li Q, Sun F, He C, Li Y, Zhou X, Zhang H, Liu X, Bu X, Gao P, He H, Zhao Z, Zhu Z. Activation of Glucagon-Like Peptide-1 Receptor Ameliorates Cognitive Decline in Type 2 Diabetes Mellitus Through a Metabolism-Independent Pathway. J Am Heart Assoc. 2021 Jul 20;10(14):e020734. doi: 10.1161/JAHA.120.020734. Epub 2021 Jul 10. PMID: 34250817; PMCID: PMC8483500.
55. Perna S, Mainardi M, Astrone P, Gozzer C, Biava A, Bacchio R, Spadaccini D, Solerte SB, Rondanelli M. 12-month effects of incretins versus SGLT2-Inhibitors on cognitive performance and metabolic profile. A randomized clinical trial in the elderly with Type-2 diabetes mellitus. Clin Pharmacol. 2018 Oct 9;10:141-151. doi: 10.2147/CPAA.S164785. PMID: 30349407; PMCID: PMC6186903.
56. Vadini F, Simeone PG, Boccatonda A, Guagnano MT, Liani R, Tripaldi R, Di Castelnuovo A, Cipollone F, Consoli A, Santilli F. Liraglutide improves memory in obese patients with prediabetes or early type 2 diabetes: a randomized, controlled study. Int J Obes (Lond). 2020 Jun;44(6):1254-1263. doi: 10.1038/s41366-020-0535-5. Epub 2020 Jan 21. PMID: 31965072.

Supplementary Table S3. Outcome-level characteristics and rationale for selection of cognitive outcomes included in the quantitative analyses

Cognitive domain	Study	Instrument	Follow-up used	Analytical sample size, GLP-1RA / control	Type of estimate used	Rationale for outcome selection
**Global cognition**	Cukierman-Yaffe et al. (2020) [24]	Montreal Cognitive Assessment (MoCA)	24 months	4351 / 4245	Intermediate post-intervention values; Hedges' g calculated from arm-level means and SDs	MoCA directly assesses global cognitive function. The 24-month scheduled assessment provided the time point most in line with the study final time point.
**Global cognition**	Dei Cas et al. (2024) [13]	Mini-Mental State Examination (MMSE)	8 months	17 / 14	Final post-intervention values; Hedges' g calculated from arm-level means and SDs	MMSE directly assesses global cognitive function.
**Global cognition**	Mullins et al. (2019) [12]	Mini-Mental State Examination (MMSE)	18 months	13 / 14	Final post-intervention values; Hedges' g calculated from arm-level means and SDs	MMSE was selected as the measure of global cognition.
**Attention**	Cukierman-Yaffe et al. (2020) [24]	Digit Symbol Substitution Test (DSST)	24 months	4336 / 4232	Intermediate post-intervention values; Hedges' g calculated from arm-level means and SDs	DSST assesses the attention/processing-speed domain. The 24-month scheduled assessment provided the time point most in line with other study final time point.
**Attention**	Cheng et al. (2022) [23]	RBANS Attention Index	4 months	12 / 12	Final post-intervention values; Hedges' g calculated from	The RBANS Attention Index directly assesses the cognitive domain of interest.

					arm-level means and SDs	
**Attention**	Luchsinger et al. (2025) [22]	Digit Symbol Substitution Test (DSST)	4 years	937/913	None	DSST was the prespecified primary cognitive outcome of the trial and directly represented the executive/attention-related cognitive domain considered in the review. The outcome was retained in the systematic review but not included in the SMD analysis because the arm-level standard deviation at the selected follow-up timepoint was not reported.
**Executive function**	Cheng et al. (2022) [23]	Stroop Color-Word Test – completion time	4 months	12 / 12	Final post-intervention values; Hedges' g calculated from arm-level means and SDs	The Stroop test directly assesses executive function. The direction of the effect was reversed so that a positive effect consistently indicated better performance with GLP-1RA treatment because lower completion time indicates better performance.
**Executive function**	Mullins et al. (2019) [12]	Trail Making Test Part B (TMT-B)	18 months	NA	None	TMT-B was selected as the measure of executive function. The outcome was retained in the systematic review but not included in the SMD analysis because the arm-level standard deviation at the selected follow-up timepoint was not reported.
**Memory**	Cheng et al. (2022) [23]	RBANS Delayed Memory Index	4 months	12 / 12	Final post-intervention values; Hedges' g calculated from arm-level means and SDs	The Delayed Memory Index was selected as the direct measure of delayed memory and provided complete arm-level data suitable for standardized effect-size calculation.
**Memory**	Luchsinger et al. (2025) [22]	Spanish English Verbal Learning Test – Delayed Recall (SEVLT-DR)	4 years	937/913	None	SEVLT delayed recall directly assesses verbal memory. Delayed recall was selected to represent the memory domain. The outcome was retained in the systematic review but not included in the SMD analysis because the arm-level standard deviation at the selected follow-up timepoint was not reported.

**Memory**	Mullins et al. (2019) [12]	California Verbal Learning Test – Long Delay Free Recall (CVLT-LDFR)	18 months	NA	None	CVLT-LDFR was selected because it directly assesses delayed verbal memory. The outcome was retained in the systematic review but not included in the SMD analysis because the arm-level standard deviation at the selected follow-up timepoint was not reported.
**Verbal fluency**	Dei Cas et al. (2024) [13]	Phonemic Verbal Fluency Test	8 months	17 / 14	Final post-intervention values; Hedges' g calculated from arm-level means and SDs	Phonemic fluency directly represents the verbal-fluency domain. The 32-week assessment was selected as the longest available follow-up for this single-study outcome.
**Verbal fluency**	Mullins et al. (2019) [12]	Verbal Fluency	18 months	NA	None	The reported verbal-fluency measure was selected as the direct assessment of this domain. The outcome was retained in the systematic review but not included in the SMD analysis because the arm-level standard deviation at the selected follow-up timepoint was not reported.
